# Rare presentation of autosomal dominant polycystic kidney disease in horseshoe kidney ultrasound evaluation: a case report

**DOI:** 10.11604/pamj.2022.42.116.31632

**Published:** 2022-06-14

**Authors:** Vadlamudi Nagendra, Suresh Vasant Phatak, Rohan Kumar Singh, Shivesh Subodh Pandey, Rishabh Gupta

**Affiliations:** 1 Department of Radiodiagnosis, Jawaharlal Nehru Medical College, Sawangi (Meghe), Wardha, Maharashtra, India

**Keywords:** Polycystic kidney disease, horseshoe kidney disease, polycystic liver disease, ultrasound, case report

## Abstract

Horseshoe kidney is a renal fusion anomaly during embryogenesis and adult polycystic kidney disease is a hereditary disorder which is transmitted in autosomal dominant pattern. Polycystic and horseshoe kidney are two separate disease entities, only about 20 cases of polycystic kidney disease in horseshoe kidney disease have been described in the literature, with an incidence ranging from 1 in 134 000 to 1 in 8 000 000 live births. We are presenting ultrasound findings of a patient who was incidentally diagnosed with polycystic horseshoe kidney on routine screening.

## Introduction

The most common hereditary renal cystic disease is autosomal dominant polycystic kidney disease, a disorder characterised by the formation of renal cysts throughout the renal parenchyma and other extrarenal complications [1]. Third most common cause of end-stage kidney disease is due to Autosomal Dominant Polycystic Kidney Disease (ADPKD) [2]. Type I ADPKD is more prevalent (about 85 percent) and is caused by mutations in the PKD1 gene, whereas type II is caused by mutations in the PKD2 gene. Extrarenal manifestations of the disease include cysts in the liver, seminal vesicles, pancreas, arachnoid membrane, intracranial aneurysms, dolichoectasias, aortic root dilatation, aneurysms, mitral valve prolapse and abdominal wall hernias [1]. The most common kidney fusion anomaly is horseshoe kidney. This disease is characterised by abnormalities in renal location, rotation and vascular supply. The isthmus of the horseshoe kidney is the characteristic feature on the US scan that clinches the diagnosis [3].

## Patient and observation

**Information of the patient:** a 50-year-old man presented with nonspecific complaints of pain in the pelvic region and increased frequency of micturition since 1 year, which was insidious in onset and progressive in nature.

**Clinical results:** per abdominal examination was within normal limits. The patient was a known case of hypertension and was on antihypertensive treatment. There was no positive family history regarding renal anomalies. The patient´s serum creatinine and urea levels were within normal limits.

**Figure 1:**
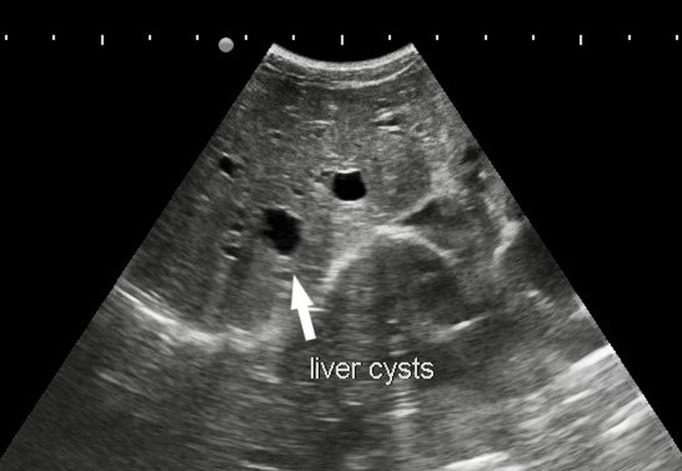
ultrasound sonography test (USG) showing empty renal fossa with non-visualisation of right kidney; multiple varied sized cysts in liver parenchyma

**Figure 2:**
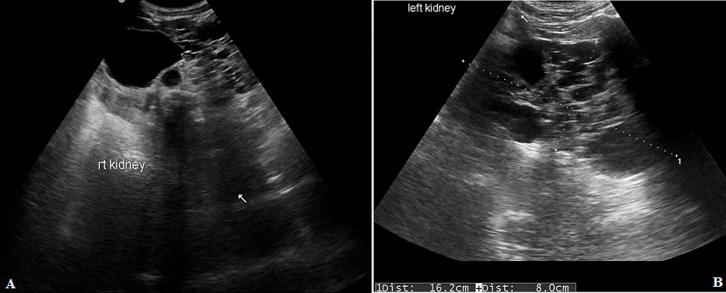
(A) USG showing right kidney which was placed low in the abdomen containing multiple anechoic cysts; (B) USG showing enlarged left kidney containing multiple anechoic cysts

**Figure 3:**
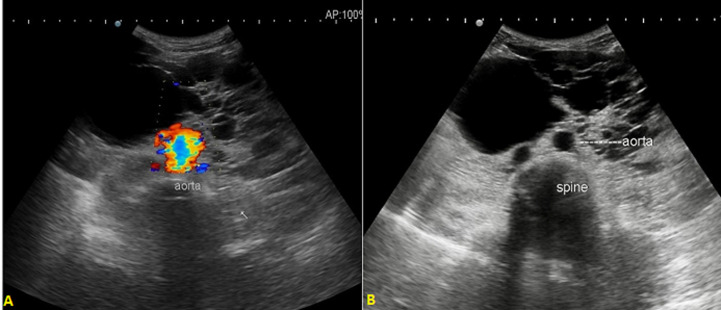
(A) USG showing isthmus which was replaced by multiple anechoic cysts connecting lower poles of bilateral kidneys; (B) isthmus was seen crossing anterior to abdominal aorta

**Diagnostic procedure:** patient was advised for sonographic evaluation for his complaints, on ultrasound abdomen, The right renal fossa was empty and liver showed multiple varied sized anechoic parenchymal cysts containing clear fluid, the largest one measuring approx. 3.2 x 2.3 cm. (Figure 1). Both kidneys were seen placed low in the abdomen, containing multiple cysts of variable sizes and causing distortion of normal architecture of kidneys (Figure 2). The longitudinal axis of both kidneys was altered and medially rotated, with an isthmus connecting the lower poles of both kidneys crossing anterior to the abdominal aorta (Figure 3). There were no calculi seen within the horseshoe kidney. The rest of the abdominal scan was within normal limits. Above ultrasound features are diagnostic of horseshoe kidney with polycystic kidney disease. The patient is from a low-socioeconomic background, so didn't have any previous access to diagnostic scans.

**Follow up:** the patient was advised three monthly abdominal ultrasound, cardiac ultrasound and kidney function test to know rate of the disease progression and to prevent complications. The patient was advised to continuously monitor his blood pressure and adhere to his antihypertensive medication.

**Consent:** patient´s written and informed consent was obtained.

## Discussion

Pathophysiology of autosomal dominant polycystic kidney - polycystin-1 and polycystin-2 are the protein products of PKD 1 and 2 genes, they are found on the epithelia of the renal tubules. Polycystin-1 is a membrane receptor that can bind and interact with a wide range of proteins, carbohydrates, and lipids, as well as elicit intracellular signals via phosphorylation pathways, whereas polycystin-2 is hypothesised to function as a calcium-permeable channel. Although the two forms of autosomal dominant polycystic kidney disease have comparable pathological and physiological characteristics, type II illness has a later start of symptoms and a slower rate of development to renal failure, providing patients a higher life expectancy (69.1 years) than type I disease (53.0 years) [4]. Because of its non-ionizing nature and low cost, renal ultrasonography is widely employed as a screening procedure. Sonographic diagnosis is done by “Ravine´s criteria” that is a patient who is at 50% risk for the disease should show at least two unilateral or bilateral cysts and should age less than 30 years; two cysts in each kidney in individuals 30-59 years; and four cysts in each kidney in individuals 60 years or older. For diagnosis, assessment, and monitoring of ADPKD, sonography is the imaging modality of choice. Gray-scale and Doppler imaging can help distinguish cysts from other solid lesions, distinguish simple from complicated cysts, and determine its vascularity. The cysts are well-circumscribed, smooth, thin-walled and show posterior enhancement on sonography [5].

Complications of ADPKD- 50% of patients aged 20 to 34 years with autosomal dominant polycystic kidney disease and with normal renal function have hypertension. Cardiovascular disease is the leading cause of mortality, so early identification and treatment of hypertension is critical. Mitral valve prolapse is the most common valvular anomaly observed on 2D echo in up to 25% of patients [1]. Poorly controlled blood pressure raises the risk of proteinuria, haematuria, renal function impairment, and morbidity and mortality from valvular heart disease and aneurysms. Despite the persistent development of cysts, renal function in most people is sustained within the normal range until 4^th^ to 6^th^ decade of life. The most frequent extrarenal manifestation is polycystic liver disease. Both PKD1 and non-PKD1 genotypes are attributed to it. Excessive growth and dilation of biliary ductules and peribiliary glands cause liver cysts. Oestrogen receptors are present in the epithelium lining hepatic cysts, and oestrogens promote the growth of hepatic cyst-derived cells. In children, hepatic cysts are uncommon, their frequency rises as one gets older. Polycystic liver disease is usually asymptomatic, but in long-term cases, symptoms might arise due to mass effect or cyst-related problems. Cysts in the pancreas are nearly asymptomatic.

Horseshoe kidney is more often asymmetrical and is left dominating (70 percent). The classic theory of mechanical fusion states that during early gestation kidneys are in the pelvis and approximate together during the metanephric stage (ie. 4^th^ week of pregnancy, 5-12 mm caudal to rostral crown-rump length (CRL)), both their lower poles come into contact and fuse in the midline, forming a horseshoe kidney with an isthmus. Another theory for horseshoe kidney development has also been linked to a teratogenic event involving the aberrant migration of posterior nephrogenic cells, which create a parenchymal isthmus. This might account for the higher risk of cancer in people who have horseshoe kidneys [3]. Over 90% of the time, kidney fusion occurs at the lower pole; however, it can also occur at the upper pole, giving an “inverted horseshoe” or at both poles, giving a “disc kidney”. The isthmus usually passes in front of the great vessels, but it can sometimes go behind them or even travel in between them [6]. From the pelvis to the mid-abdomen, the horseshoe kidney can be located anywhere along its route of normal renal ascent. Because the inferior mesentric artery prevents the renal isthmus from ascending, it is more often found in a lower lumbar region [3]. Renal vein abnormalities are common in horseshoe kidneys (23 percent). The vascular supply of the isthmus is of special significance and varies greatly; in rare situations, it may supply the whole kidney. Horse shoe kidney is also linked to a variety of ureteric arrangements and renal venous abnormalities. Therefore, contrast enhanced computed tomography (CT) is necessary, as these have significant consequences for surgical planning [6]. The isthmus contains functioning renal parenchyma in 80% of patients, making it difficult to separate securely during surgery. The most common horseshoe kidney complication is stone formation (16-60 percent of patients), due to Uretero-Pelvic junction occlusion and is further aggravated by stasis and infection [3].

Horseshoe kidney is not a clinical diagnosis, so imaging by sonography, CT, and magnetic resonance imaging (MRI) is necessary. The identification of a midline isthmus connecting the two lower poles of the kidney across the midline on ultrasonography is the key finding in making a diagnosis of a horseshoe kidney [7]. The isthmus and anatomic location of the kidneys are particularly well-detected by CT and MRI [8]. With the presence of horseshoe kidney, the likelihood of progression to end-stage renal failure in ADPKD is not known to increase, and the rationale for nephrectomy or transplant remains constant.

## Conclusion

Polycystic horseshoe kidney is a rare entity. Ultrasound and color Doppler has high accuracy for its diagnosis. It also has a role to play in assessment of disease progression and possible complications.
